# Recent Advances of Gold Compounds in Anticancer Immunity

**DOI:** 10.3389/fchem.2020.00543

**Published:** 2020-06-30

**Authors:** Shuang Yue, Miao Luo, Huiguo Liu, Shuang Wei

**Affiliations:** Department of Respiratory and Critical Care Medicine, Tongji Hospital, Tongji Medical College Huazhong University of Science and Technology, Wuhan, China

**Keywords:** gold compounds, innate immunity, adaptive immunity, immunogenic cell death, immune checkpoints

## Abstract

In recent years, gold compounds have gained more and more attentions in the design of new metal anticancer drugs. Numerous researches have reported that gold compounds, in addition to their widely studied cytotoxic antitumor effects, also reverse tumor immune escape and directly facilitate the functions of immune cells, leading to enhanced anticancer effects. This review mainly summarizes our current understandings of antitumor effects of gold drugs and their relationships with various aspects of antitumor immunity, including innate immunity, adaptive immunity, immunogenic cell death, and immune checkpoints, as well as their roles in adverse effects. Some recent examples of anticancer gold compounds are highlighted. The property of gold compounds is expected to combine with anticancer immunotherapy, such as immune checkpoint inhibitors, to develop new anticancer therapeutic strategies.

## Introduction

From the accidental discovery of cisplatin's antitumor activity by Rosenberg and his coworkers, platinum-based complexes have been used as standard chemotherapeutic agents more than 40 years in clinical practice (Rosenberg et al., 1969). However, we found that platinum-based complexes are only effective against limited types of tumors and have a variety of serious side effects (such as gastrointestinal, nervous system toxicity and bone marrow suppression) (Hartmann and Lipp, 2003; Wang and Guo, 2013; Stojanovska et al., 2015, 2017; Oun et al., 2018). Additionally, intrinsic and acquired drug resistance attenuate the effectiveness of these agents (Martinez-Balibrea et al., 2015). For this reason, more efforts are urgently needed to explore novel anti-tumor metallodrugs to substitute the widely used platinum complexes. Many new metal complexes have been reported to have antitumor effects including gold, silver, copper, ruthenium and other active metals. Among them, coinage metals (especially Au and Ag) have shown greater application potential because they are less toxic to human body than other transition metals. Gold compounds deserve particular attention, from the view of chemical, because the unique position of gold in the periodic table, which ultimately leads to the highest electronegativity, electron affinity as well as redox potential compared to other metals. Gold compounds exert cytotoxic effects by inhibiting thiol-containing enzymes, especially TrxR (Liu and Gust, 2013; Ortego et al., 2014; Bian et al., 2019, 2020a,b; Fan et al., 2019), damaging mitochondrial (Rigobello et al., 2002; Rackham et al., 2007) and DNA function (Messori et al., 2005; Patel et al., 2013), all of which may contribute to their clinical anticancer activity. Recently, many groups have found that auranofin, a gold compound widely used in antirheumatic therapy (Sadler and Sue, 1994; Shaw, 1999), also has anticancer, antibacterial and other properties (Marzano et al., 2007; Fiskus et al., 2014; Harbut et al., 2015; Diez-Martinez et al., 2016; Thangamani et al., 2017; AbdelKhalek et al., 2019; Onodera et al., 2019; Raninga et al., 2020). Therefore, there is growing interest in the investigation of gold compounds with new applications. Although no non-platinum metal compounds have been approved for cancer treatment, a number of gold drug candidates are being considered. Some novel gold compounds have shown promising results in preclinical researches (Ott and Gust, 2007).

Cancer immunotherapy is a promising research field and is gaining more and more attention from the scientific community. Recent immune checkpoint inhibitors are starting a golden age of tumor immunotherapy. In the early days, based on clinically observed chemotherapy-induced myelosuppression and lymphocytopenia (Grossman et al., 2015; Cao et al., 2016; Kamimura et al., 2016; Oun et al., 2018), it is taken for granted that the primarily effect of chemotherapy on the immune system is immunosuppression. Interestingly, Taro Shimizu and his coworkers (Shimizu et al., 2017) found that liposomal oxaliplatin could significantly suppress the growth of neoplasms implanted in immunocompetent murine, but not in immunodeficient murine. The phenomenon was also observed in other groups using different mice tumor models and mice strains with metal-based compounds (Tesniere et al., 2010; Jungwirth et al., 2012; Chang et al., 2013). Hence, we propose a hypothesis that in addition to the classical DNA damage pattern of platinum complexes, the immune system may increase the antitumor activity of these drugs in a synergistic manner. Furthermore, although gold is clinically used for immune suppression (in rheumatoid arthritis), it can also produce toxicities resulting from immune stimulation (Merchant, 1998). The use of gold drugs is often accompanied by adverse immune reactions including diverse forms of dermatitis, glomerulonephritis, cytopenias, hepatitis and pneumonitis (Havarinasab et al., 2007). A series of literatures have shown that gold compounds may stimulate an anticancer immune response. What are the complicated interactions between gold compounds and the immune system? However, The intricate interrelationships of gold compounds with the immune system and the underlying molecular biological mechanisms are unclear.

Given the revolutionary achievement of platinum-based complexes, it is not surprising that the field of inorganic medicinal chemistry has been predominated by researches on the antitumor activity of metal complexes. Particularly, the preparation of novel gold compounds for cancer therapy has been accelerating in recent decades, and a large number of research reports are published every year. In this review, we aim to summarize the complex relationship between various gold derivatives and immune system, and the role of immune system in their anticancer activity as well as adverse effects, in order to explore their novel applications in cancer combination immunotherapy.

## Immune Surveillance and Immune Evasion

Before introducing the anticancer immune activity of gold compounds, an overview on the general aspects of the body's immune response and the main players in the immune system are given in the following.

**Figure 1:**
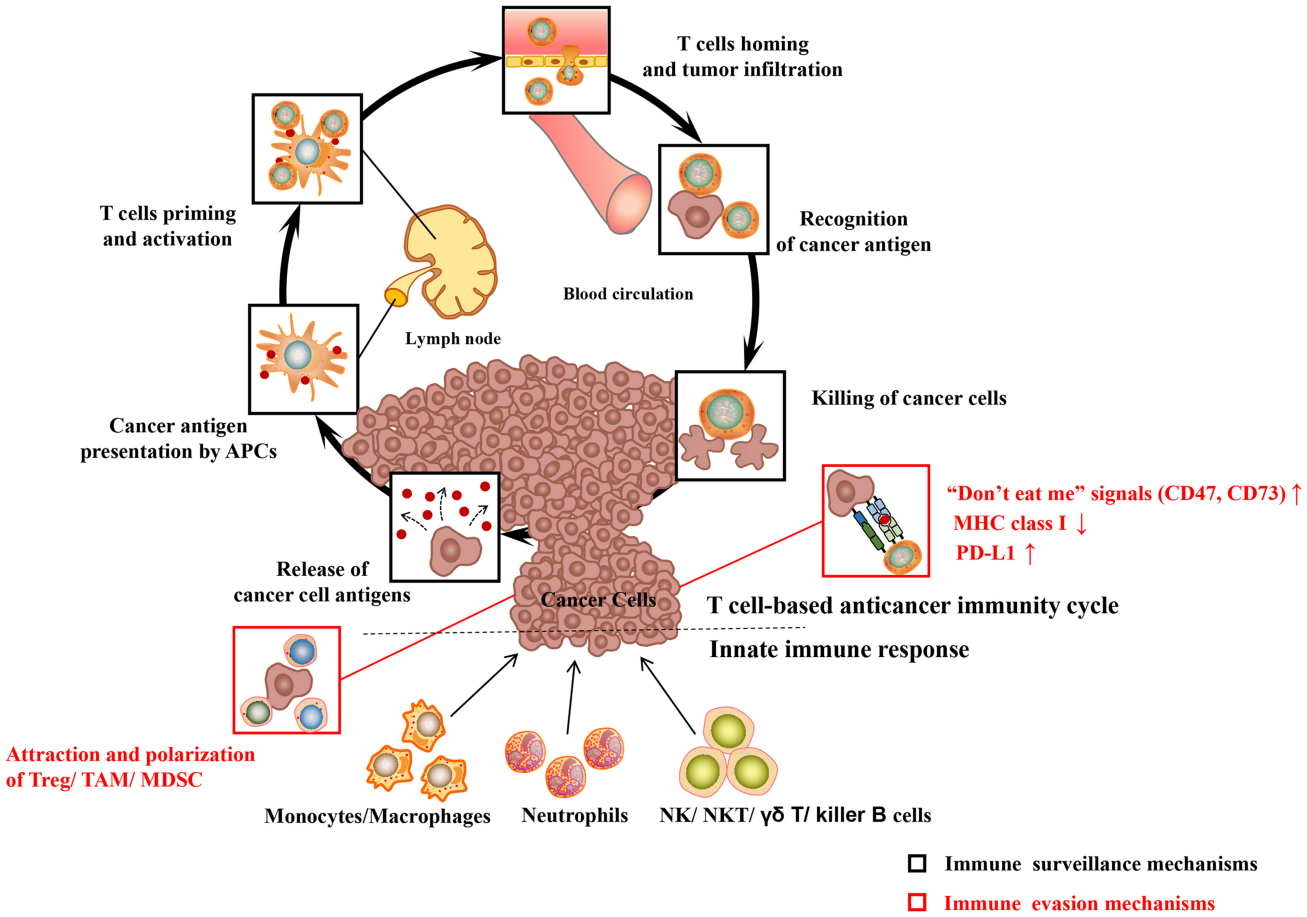
Major cancer immune surveillance and immune evasion mechanisms. For detailed description see text. APCs, antigen presenting cells; MHC class I, major histocompatibility complex class I; PD-L1, programmed death-ligand 1; Treg, regulatory T cell; MDSC, myeloid-derived suppressor cell; TAM, tumor-associated macrophages; NK cell, natural killer cell; NKT cell, natural killer T cell. Adapted from Chen and Mellman (2013).

The body's immune system is equipped with elaborate innate and adaptive immune mechanisms devoted to effectively recognize and eliminate pathogens as well as preventing malignant transformation (“immune surveillance”) (see Figure 1) (Chen and Mellman, 2013). Innate immunity is the body's first line of defense against foreign pathogens invasion, which is a rapid non-specific immune response to pathogens. Prominent among these are monocytes/macrophages, neutrophilic granulocytes (neutrophils), natural killing (NK) cells as well as dendritic cells (DC). During their patrols, innate immune cells may sense changes of molecular patterns in the malignant tissue, which is called as damage-associated molecular patterns (DAMP) or alarmins. Pattern recognition receptors (PRR) on the surface of immune cells mainly include the toll-like receptors (TLRs), which mediate the recognition, killing or phagocytosis of abnormal cells. At the same time, both are accompanied by immune-stimulating inflammation. In addition to PRR, the phagocytosis of cancer cells may be triggered by the opsonization of antibodies and complement on the cell surface. The T cell anticancer immune cycle is a process of self-proliferation and self-amplification, which connects innate immunity and adaptive immunity in a highly complex fashion, leading to antigen-specific T cell-mediated immune response. Professional antigen presenting cells (APC), especially DC, play a key role in activating specific anticancer immune responses. When they patrol the (pre)malignant tissue, they may sense a change of molecular patterns known as so-called DAMP. The activated DC migrates to the tumor draining lymph nodes and presents tumor-specific antigens to naive CD8 + or CD4 + T cells by MHC class I or MHC class II molecules, respectively (“first” signal). Additionally, under the action of costimulatory signals (“second” signal, including the coaction of B7-type CD80/86 receptor on DC with CD28 on T lymphocytes) and cytokines (“third” signal, such as IL-2 and IL-12), activated T lymphocytes travel to the (pre)neoplastic tissue through the bloodstream, and followed by specific destruction of (pre)neoplastic cells expressing the respective tumor-specific antigens.

However, cancer cells have evolved multiple immune-regulatory mechanisms to evade recognition and destruction of the innate and adaptive immune systems, leading to the development of clinical tumors (“immune escape”) (see Figure 1). For example, cancer cells may elude recognition of innate immune cells (including macrophages, neutrophils) by upregulating “don't eat me” signals such as CD47 or CD73 (Chao et al., 2010). Malignant cancer cells can evade the recognition and destruction of cytotoxic T cells (CD8+T cells or CTL) by down-regulating the expression of MHC class I molecules (Bradley et al., 2015). More recently, the perspective of immune checkpoints has been proposed, including programmed-death/programmed-death ligand 1 (PD1/PD-L1) system, cytotoxic T-lymphocyte-associated protein 4 (CTLA-4). Blocking inhibitory receptors or ligands through immune checkpoint inhibitors is one of the most successful and promising anticancer immunotherapy strategies so far. The PD-L1 expressed on the surface of tumor cells is able to combine with the inactivating receptor PD1 on adaptive immune T cells, thus leading to their deactivation and CTL anergy. A large number of researches indicate that the high expression of PD-*L1* on cancer cells leads to the anergy and exhaustion of T cells, which restricts CTL from effectively targeting cancer cells (Zhang et al., 2018a). In the development of cancer, tumor cells can attract regulatory immune cells (including regulatory T cell, tumor-associated macrophages) to the tumor microenvironment (TME), which is dominated by immunosuppressive myeloid cell types, through the production of immunosuppressive chemokines (such as CCL2) (Muenst et al., 2016). In addition, cancer cells can induce the polarization of immune cells into immunosuppressive phenotypes, such as M2 macrophages (via CSF-1), TH 2 cells, and regulatory T cells (via TGF-β, IL-10) (Murray et al., 2014; Noy and Pollard, 2014). In conclusion, cancer cells have evolved multiple ways to escape the recognition and damage of innate and adaptive immune systems. Hence, more efforts have been focused on enhancing the body's anti-tumor immune response with regard to the current cancer immunotherapy.

A lot of evidence from experimental and clinical studies suggest that the antitumor mechanisms of gold compounds are extremely complex and diverse. Recently, it has been reported that gold compounds may have a potential relationship with anti-tumor immunity. Gold chemotherapy drugs, in addition to their widely studied cytotoxic antitumor effects, might reverse important aspects of tumor immune escape and directly affect several types of immune cells, leading to enhanced anticancer effects. In this article, we will summarize the relationship between gold compounds and various aspects of antitumor immunity, including innate immunity, adaptive immunity, immunogenic cell death, and immune checkpoints.

## Antitumor Immune Effects of Gold Compounds

### Gold Compounds and Innate Immune System

**Figure 2:**
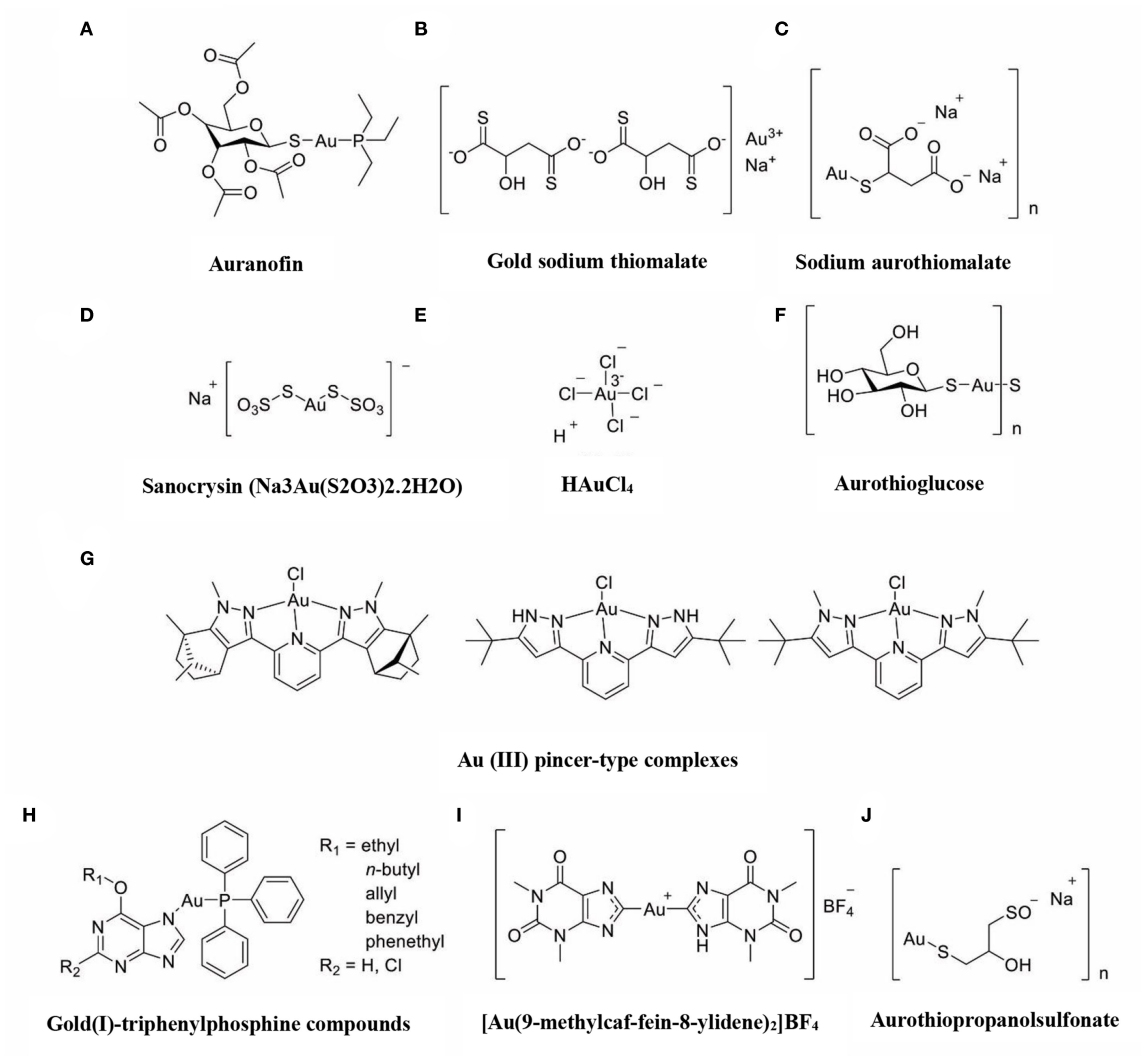
The chemical structure of several common gold compounds. **(A)** Auranofin; **(B)** Gold sodium thiomalate; **(C)** Sodium aurothiomalate; **(D)** Sanocrysin Na_3_Au (S_2_O_3_)_2_·2H_2_O; **(E)** HAuCL4; **(F)** Aurothioglucose; **(G)** Au(III) pincer-type complexes; **(H)** Gold(I)-triphenylphosphine compounds; **(I)** [Au(9-methylcaf-fein-8- ylidene)2]BF4; **(J)** Aurothiopropanolsulfonate.

**Figure 3:**
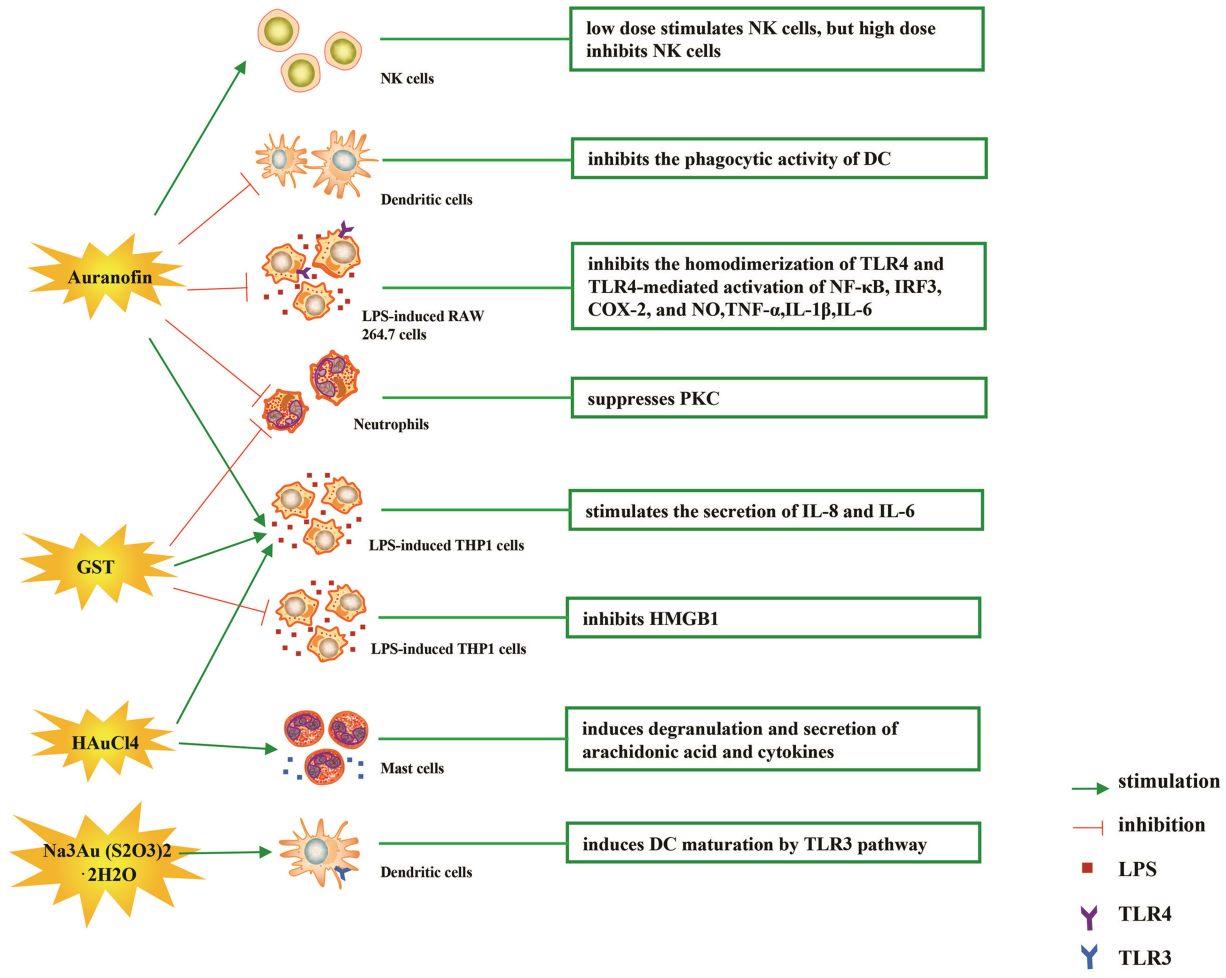
Effects of several major gold complexes on innate immune system. gold compounds can not only promote direct immune cell-mediated destruction, but also synergistic promote the T cell-based anticancer immunity cycle via DC. Gold complexes can induce the activation and proliferation of NK, mast cells, monocytes/macrophages, neutrophils, and release of various inflammatory mediators. Furthermore, gold complexes induce DC maturation and enhance antigen presentation through TLR3 dependent signaling. For detailed description see text. NK cell, natural killer cell; DC, dendritic cell; LPS, lipopolysaccharide; TLR, toll-like receptor; TNF-α, tumor necrosis factor α; IL, interleukin; AuTM, sodium aurothiomalate; GST, gold sodium thiomalate; PKC, protein kinase C; HMGB1, high mobility group box chromosomal protein 1.

The immune-regulatory effects of gold drugs have been comprehensively reviewed in the literatures concerning rheumatoid arthritis as well as other immune-related diseases, such as HIV and malaria (Griem and Gleichmann, 1996; Madeira et al., 2012; Nardon et al., 2016). Figure 2 shows the chemical structure of several common gold compounds. Several common metals (such as gold, nickel, copper, and mercury) have been found to have the ability to stimulate innate immunity (Suzuki et al., 2011; Rachmawati et al., 2015a). A series of *in vitro* and *in vivo* studies have shown that gold compounds can not only promote direct immune cell-mediated destruction, but also synergistic promote the T cell-based anticancer immunity cycle via DC (see Figure 3). The complex mechanisms of the effects of gold compounds on the innate immune system are summarized in Table 1. Gold compounds can induce cancer cells destruction through various forms of cytotoxic action, leading to the expression of proteins on the cell surface, secretion of cytokines, or rupture of the plasma membrane and release of intracellular substances. Released cytoplasmic molecules are danger signals, known as DAMPs, which enable the immune system more sensitive to the recognition of tumor antigens. Rachmawati et al. reported that the gold compound (Na_3_Au (S_2_O_3_)2·_2_H_2_O, Figure 2D) induced substantial release of the pro-inflammatory mediator IL-8 from DC, PBMC, and THP-1 cells and expression of CD40 on the surface of DC, indicating DC's maturation and adaptive immune stimulatory capacity. The ability of this gold compound to induce innate immune responses can be attributed to TLR3 dependent signaling (Rachmawati et al., 2015b). I. Stern et al. observed the effects of auranofin (Figure 2A), gold sodium thiomalate (Figure 2C), and HAuCl4 [Au (III)] (Figure 2E) on the ability of LPS-induced THP1 monocytes to secrete key inflammatory cytokines (IL6, IL8, IL10, and TNF α) *in vitro*. The results showed that sub-lethal concentrations of the three gold compounds could differentially modulate activation of monocytes. Among them, the effect of auranofin was slightly stronger (Stern et al., 2005). Another study suggested that the activation of monocytes may be related to the success of gold compounds in the treatment of rheumatoid arthritis (Hurst et al., 1986). Mast cells play key roles in allergic and inflammatory responses. There is considerable evidence that mast cells are important for adaptive and innate immunity (Galli et al., 2005), as well as the development of autoimmune diseases (Costela-Ruiz et al., 2018). In a previous review, gold, mercury, and silver all had effects on mast cell signaling, function, and survival, inducing aberrant immunological responses. All these compounds stimulated mast cells to degranulate and secrete arachidonic acid metabolites as well as cytokines such as interleukin-4 (Suzuki et al., 2011). HAuCl4 [Au (III)] at a non-toxic concentration ( ≤ 50 μM) stimulated large amounts of degranulation and leukotriene C4 secretion in mast cells by a Ca2^+^-dependent manner (Hayama et al., 2011). It has been reported that auranofin has a dose-dependent bidirectional effect on NK cell activity, enhancing NK cell activity at low dose and inhibiting NK cell activity at high dose *in vitro* (Russell et al., 1982; Pedersen and Abom, 1986). However, as for TLR signaling, Youn et al. found that auranofin suppressed LPS-induced homodimerization of TLR4 and TLR4-mediated activation of key transcription factors (such as NF-κB, IRF3, and COX-2) in mice pro-B as well as monocytic cell lines. In addition, auranofin also inhibited NF-κB activation induced by MyD88-dependent downstream signaling elements of TLR4, MyD88, IKKβ, and p65 (Youn et al., 2006). Auranofin suppressed multiple steps in TLR4 and downstream signaling, thereby inhibiting immune inflammation. The experimental results of Wang et al. showed that disodium aurothiomalate could inhibit the activity of CD45, a protein-tyrosine phosphatase expressed on all white blood cells, which could enhance the signaling of B and T cell antigen receptors (Wang et al., 1997).

**Table 1:** Effects of gold compounds on innate immune system.

**Gold compounds**	**Mechanism of action**	**Cell or animal models**	**Reference**
Auranofin	Low dose stimulates NK cells, but high dose inhibits NK cells	NK cells	Russell et al., 1982; Pedersen and Abom, 1986
	Inhibits the homodimerization of TLR4 and TLR4-mediated activation of key transcription factors (NF-κB, IRF3, COX-2)	Murine pro-B, RAW264.7, 293T, COS-7cells	Jeon et al., 2003; Youn et al., 2006
	Suppresses PKC	Human neutrophils	Parente et al., 1989
	Activates monocytes to secrete key inflammatory cytokines (IL6, IL8)	THP1 cells	Stern et al., 2005
	Inhibits NO and pro-inflammatory cytokines (TNF-α, IL-1β and IL-6)	RAW 264.7	Han et al., 2008a
GST	Activates monocytes to secrete key inflammatory cytokines (IL6, IL8)	THP1 cells	Stern et al., 2005
	Inhibits PKC, HMGB1 translocation, IFN-beta, NO, and the release of HMGB1	RAW 264.7, THP-1 cells	Parente et al., 1989; Zetterstrom et al., 2008
AuTM	Activates monocytes and enhances release of superoxide	Human monocytes	Hurst et al., 1986
	Inhibits CD45		Wang et al., 1997
Na_3_Au (S_2_O_3_)_2_·2H_2_O	Induces release of IL-8, and expression of CD40 on the surface of DC	DC, PBMC, THP1 cells	Rachmawati et al., 2015b
HAuCl4 [Au(III)]	Stimulates monocytes to secrete IL6, IL8, and mast cells to degranulate and secrete arachidonic acid metabolites and cytokines (such as IL-4)	THP1, RBL-2H3, HMC-1 cells	Stern et al., 2005; Hayama et al., 2011; Suzuki et al., 2011
Aurothioglucose	Inhibits the DNA binding activity of NF-κB		Yang et al., 1995
Gold(I)-triphenylphosphine compounds	Inhibits pro-inflammatory cytokines (TNF-α IL-1β)	THP-1 cells	Krikavova et al., 2014

*AuTM, sodium aurothiomalate; GST, gold sodium thiomalate; NK cell, natural killer cell; DC, dendritic cell; TLR, toll-like receptor; TNF-α, tumor necrosis factor α; HMGB1, high mobility group box chromosomal protein 1; IL, interleukin; PKC, protein kinase C; IFN, interferon*.

Gold compounds have also been shown to affect some important signaling pathways and key transcription factors. The NF-κB and protein kinase C (PKC) signaling pathways play a key role in the process of activation, differentiation, and maturation of myeloid and lymphatic cells. NF-κB is a key transcription factor involved in the expression of many inflammatory genes and plays an important role in oncogenesis, which is associated with the proliferation of multiple types of tumors (Zeligs et al., 2016). There are growing interests in exploring novel regulators to inhibit NF-κB activation, because blocking different steps of NF-κB signaling pathway may slow tumor growth, progression, and chemotherapy resistance. Auranofin suppresses nuclear translocation of NF-κB by blocking IκB kinase (IKK) activation in macrophages. This inhibitory activity may be related to the suppression of TNF-α (Jeon et al., 2000). It also has been reported that auranofin inhibits NF-κB activation by modifying Cys-179 of IKKβ subunit in monkey kidney (COS-7) cells as well as LPS-stimulated macrophages (Jeon et al., 2003). Aurothioglucose (Figure 2F) has a strong inhibitory effect on the DNA binding activity of NF-κB, which is essential for its performance (Yang et al., 1995). The PKC activity was shown to be inhibited by auranofin as well as gold sodium thiomalate in human neutrophils (Parente et al., 1989).

However, gold (I) compounds have been clinically used to treat rheumatoid arthritis due to their anti-inflammatory properties, which appears contradictory to its immune stimulatory activity. This intrigues clinicians and toxicologists for many years. Anyway, the gold paradox is not unique, steroids are also known to have both immunostimulatory and immunosuppressive effects. Radka et al. reported a series of gold(I)-triphenylphosphine compounds (Figure 2H) with hypoxanthine-derived ligands. These complexes inhibited the secretion of pro-inflammatory cytokines, such as tumor necrosis factor-α (TNF-α) and interleukin-1β (IL-1β), in the lipopolysaccharide-activated macrophage-like THP-1 cell model (Krikavova et al., 2014). Gold sodium thiomalate (GST, Figure 2B) inhibited release of the key endogenous mediators of HMGB1 translocation, IFN-beta and NO, thus reducing the extracellular release of HMGB1 in murine RAW 264.7 and human THP-1 macrophages *in vitro* (Zetterstrom et al., 2008). The differential effects of gold compounds on the immune system may be related to drug dose, duration, cell lines, ligand composition, as well as the oxidation state of Au. The differences in the patterns of action of gold compounds between inflammation and cancer are not clear. The suppression of cancer-promoting inflammation has been presumed as one of the main mechanisms in the antitumor activity of gold compounds (Madeira et al., 2012). The anti-inflammatory and anticancer activities of gold compounds may involve similar cytokines and molecular pathways but the degree of induction varies (Yamamoto and Gaynor, 2001). In addition to the inhibition of TLR signaling and NF-κB signaling pathway mentioned above, auranofin also reduces TNF-α synthesis and secretion, decreases STAT-3 transcription activity as well as inhibits angiogenesis (Kim et al., 2007; Han et al., 2008a), which are related to tumor growth and development. Pro-inflammatory cytokines like interleukins (IL) are wildly known to be related to malignant progression and metastasis in multiple types of tumors, by regulating the expression of matrix metalloproteinases (MMP) and angiogenic proteins growth factors including VEGF (Quail and Joyce, 2013). Auranofin and the heterobimetallic Ru-Au compound (RANCE-1) have a strong inhibition of several cytokines (IL-6, IL-5, IL17A, and IL-8) in Caki-1 renal cancer cells (Elie et al., 2018). Auranofin also decreased the production of nitric oxide (NO) as well as the pro-inflammatory cytokines (TNF-α, IL-1β and IL-6) in macrophages (Han et al., 2008a). The development and evolution of tumors are associated with a series of inflammatory pathways. The blocking of several inflammatory pathways associated with tumor development has a promising potential to promote its anticancer activity, although the direct effects of gold compounds on innate immune cells have been little studied. Given the promising anticancer activity of gold compounds in many types of tumors, the role of immuno-regulatory and anti-inflammatory effects needs to be further investigated at the experimental level.

### Gold Compounds and Adaptive Immune System

**Figure 4:**
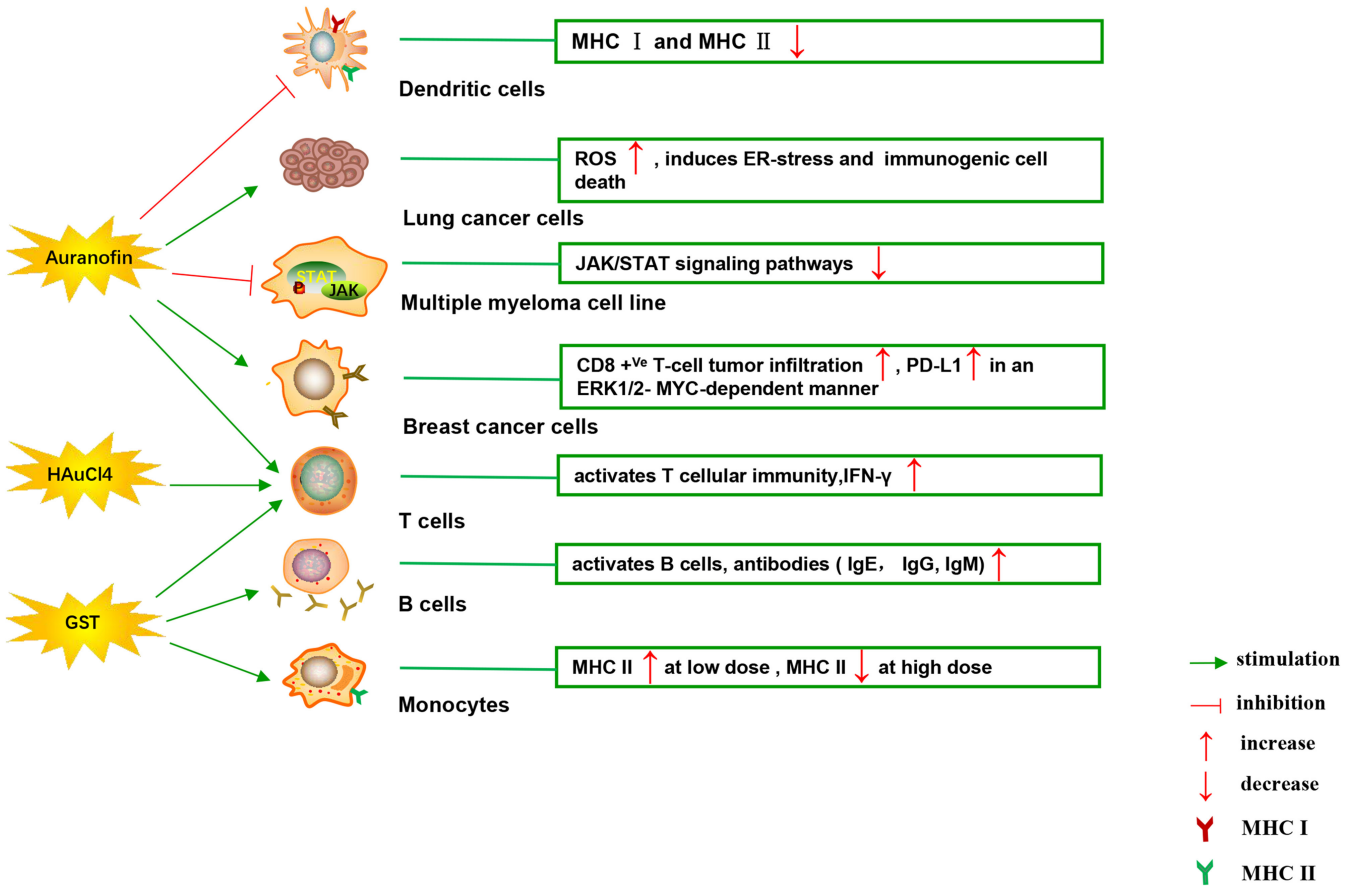
Effects of several major gold complexes on adaptive immune system. Gold compounds stimulate the activation and proliferation of B cells and T cells. Antigen presenting cells (mainly dendritic cells) presents tumor antigens to T lymphocytes to initiate antigen-specific T cell immune responses. Gold derivatives can regulate the level of MHC class I and MHC class II molecules, and up-regulate the expression of PD-L1 on the surface of cancer cells. For detailed description see text. MHC, major histocompatibility complex; ROS, reactive oxygen species; ER-stress, endoplasmic reticulum stress; PD-L1, programmed death-ligand 1; GST, gold sodium thiomalate.

Figure 4 illustrates various aspects of the effect of gold compounds on the adaptive immune system. Gold compounds can enhance the antigenicity of cancer cells. The body's adaptive immune system is able to identify “non-self” antigens through MHC class I presentation of mutation derived immunogenic neoantigens or viral peptides (in virus-induced tumor) by the malignant cells. This is closely related to anticancer metal chemotherapy drugs which are believed to have mutagenesis in many cases. Indeed, the increased number of mutations as well as translocations would support adaptive immune system to identify “non-self” malignant neoantigens (Siniard and Harada, 2017). Although the interaction between gold-based drugs and DNA is weaker than that of platinum, they also induce some mutations. A panel of new Au (III) complexes with pincer type ligands (Figure 2G) had moderate binding affinity with calf thymus DNA (CT DNA), and molecular docking showed that they interacted with DNA by insertion (Radisavljevic et al., 2018). By a joint ESI MS and X ray diffraction (XRD) methods, the dicarbene gold(I) compound [Au(9-methylcaf-fein-8-ylidene)2] BF4 (Figure 2I) had a tight binding to Tel 23 DNA G-quadruplex (telomeric nucleic acid sequences with rich guanines) and formed stable adducts (Bazzicalupi et al., 2016). The complex interactions between gold compounds and DNA, including insertion, covalent binding, and even unobserved ways of acting, generate a number of damaged DNA cells and even induce the DNA mutation when the self-repair mechanisms dysfunction, thus exposing more neoantigens. According to a research by Kazuo and his co-workers, pretreatment with Au(III) complex of a model protein antigen (bovine ribonuclease A) induced novel antigen peptide recognized by CD4 ^+^ T cells (Takahashi et al., 1994) (Table 2).

**Table 2:** Effects of gold compounds on adaptive immune system.

**Gold compounds**	**Mechanism of action**	**Cell or animal models**	**Reference**
Auranofin	Stimulates T effector and T suppressor lymphocytes	C57BI mice	Walz and Griswold, 1978
	Inhibits MHC class I and MHC class II	DC2.4 cells, BM-derived DCs	Han et al., 2008a
Au (III) pincer-type complexes	Insert DNA and interfere with the cell cycle	A549, A375, and LS-174 cells	Radisavljevic et al., 2018
Dicarbene gold(I) complex	Forms stable adducts with Tel 23 DNA G-quadruplex		Bazzicalupi et al., 2016
GST	Activates B cell and antibody (IgE, IgG, IgM) production	BALBc, C_s_H, C57BI, AKR mice	Measel, 1975; Havarinasab et al., 2007
	Stimulates T effector and T suppressor lymphocytes	C57BI mice	Walz and Griswold, 1978
	Low dose promotes MHC II, but high dose inhibits MHC II	human monocytes	Sanders et al., 1987
Aurothiopropanolsulfonate	Enhances the antigen-specific IgE immune response	Brown-Norway (BN) rats	Kermarrec et al., 1996
HAuCl_4_	Activates CD4+T and CD8+T cell, and promotes release of IFN-γ	BN rats	Savignac et al., 2001
Gold nanoparticles	Promotes the immunogenicity of antigens, DC maturation, TLR9 expression, memory CD8+T cells activation, MHC II and CD 86 expression	BMDCs, and RAW264.7, GMK-AH1, JAWSII cells	Niikura et al., 2013; Orlowski et al., 2018

*MHC, major histocompatibility complex; DC, dendritic cell; GST, gold sodium thiomalate; IFN-γ, interferon-γ; TLR, toll-like receptor*.

Interestingly, gold compounds also stimulate the activation and proliferation of T as well as B cells (see Table 2). The mice treated with organic gold compounds showed immune stimulation, which was manifested as plaque-forming cells, rosette-forming cells, and serum antibody elevation (Measel, 1975). Some studies indicated that aurothiopropanolsulfonate (Figure 2J) could also enhance the antigen-specific IgE immune response in mice (Kermarrec et al., 1996). Gold, in the form of natrium aurothiomaleate (GSTM), had a strong B cell-stimulating effect, including T helper 1 (Th1)- and Th2- isotypes (Havarinasab et al., 2007). Moreover, Walz et al. found that the stimulatory effects of auranofin and GST on cell-mediated immunity as evidenced by oxazolone-induced contact sensitivity as well as delayed hypersensitivity to sheep red blood cells (DH-SRBC). Their results showed that gold in the form of auranofin was approximately 4 times more effective in stimulating cellular immunity than gold in the form of GST (Walz and Griswold, 1978). Gold salt (HAuCl4) stimulated CD4 ^+^ T and CD8 ^+^ T cell activation as well as promoted the secretion of IFN-γ in rats (Savignac et al., 2001). What's more, disodium aurothiomalate interfered with antigen presentation and CD4 ^+^ T cell activation by binding peptides containing cysteine residues (Griem et al., 1995). GST preferentially inhibited B cells at much lower concentrations than required for T cells interference (Hirohata, 1996).

Furthermore, it is well-known that cancer cells tend to reduce MHC class I expression to prevent tumor-specific CTL clone cells from recognizing neoantigens or “tumor-associated antigens” (TAA) during immune evasion. Several researches have shown that metal-based treatment (like platinum) might increase the expression of MHC class I in cancer cells (Ohtsukasa et al., 2003; Gameiro et al., 2012). To our surprise, there is little research with regard to the specific effects of gold compounds on MHC expression. It's reported that GST stimulated the up-regulation of MHC class II expression at low concentration, but it was inhibited at high concentration (Sanders et al., 1987). Auranofin inhibited MHC class I and MHC class II-restricted antigen presentation in dendritic cells of mice (Han et al., 2008b). In addition, various papers have showed that metal nanoparticles (NPs) including gold, silver, nickel, and iron oxide are able to increase the immunogenicity of antigens (Niikura et al., 2013; Orlowski et al., 2018). A study by Piotr and his co-workers demonstrated that tannic acid-modified silver and gold nanoparticles (TA-Ag/AuNPs) stimulated DCs maturation, TLR9 expression and memory CD8+T cells activation, and helped to overcome virus-induced suppression of DCs activation by up-regulation of MHC II and CD 86 expression (Orlowski et al., 2018). We speculate that gold compounds can also enhance antigen presentation by up-regulating MHC expression, which requires more efforts to explore. All of this suggests that gold is a promising immune-regulator whose multiple effects on the adaptive immunity remain to be discovered (Table 2).

### Gold Compounds Induce Immunogenic Cell Death

It is currently accepted that, in certain settings, chemotherapy drugs are able to activate the entire anticancer immune cycle and even cause a persistent immunological anticancer memory by inducing tumor cells death. This ideal form of tumor chemotherapy-induced cell death is known as immunogenic cell death (ICD) (Englinger et al., 2019). The conception of ICD was first proposed in 2005 in the context of antitumor chemotherapy, and based on the observation that mice colon tumor cells with anthracycline doxorubicin treatment *in vitro* were able to induce an effective anticancer vaccination reaction that inhibited the growth of inoculated cancers or caused the regression of the established tumor (Casares et al., 2005). Under normal physiological conditions, the process of apoptosis is immunologically “silent” and includes an efficient engulfment by phagocytes to prevent the release of intracellular components that activate inflammation and autoimmune responses. In contrast, ICD in cancer therapy induces immune-stimulatory rather than immunosuppressive reaction. ICD is able to reverse several crucial aspects of immune evasion, thus re-inducing the immune recognition of tumor cells. Generally, apoptotic cancer cells expose “find me” and “eat me” signals to attract innate immune cells (especially DCs), leading to the phagocytosis of apoptotic cells and antigen presentation. The initiation of ICD is based on several different molecular mechanisms in the dying tumor cells, mainly by endoplasmic reticulum (ER) stress- and autophagy-mediated release of DAMP molecules, including the protein chaperones calreticulin (CRT), the nucleotide ATP, DNA damage sensitizer HMGB1, CXCL10 as well as HSP70/90 (Terenzi et al., 2016).

The generation of ER stress mediated by ROS is the core of ICD induction, thus leading to the classification of ICD inducers into two categories. Type I ICD inducers exert multiple cytotoxic effects and trigger ER stress as a secondary mode of action, while type II inducers mediates ROS-related ER-stress as the main mechanism of action (Garg et al., 2015). By far most of the ICD inducers used in clinical anticancer chemotherapy belong to type I class, such as anthracyclines and mitoxantrone, the glycopeptide bleomycin, the alkylating agent cyclophosphamide, bortezomib, as well as the metal-based agent oxaliplatin (Tesniere et al., 2010; Kroemer et al., 2013; Sun et al., 2019; Zhou et al., 2019). Although type II ICD inducers are rare, they have recently been identified in preclinical metal-containing compounds (Wong et al., 2015). Although some similarities between oxaliplatin and cisplatin, it is surprising that only the former has been reported as a true ICD inducer (type I) (Tesniere et al., 2010). The specific relationship between the ICD-inducing capacity of metal complexes and their structure and mode of action is unclear. Interestingly, multiple antitumor metal drugs, such as gold (Marmol et al., 2017; Huang et al., 2018), copper (Bortolozzi et al., 2014; Yang et al., 2017), iron (Liu and Connor, 2012; Kim et al., 2016), arsenic (Chiu et al., 2015), ruthenium (Flocke et al., 2016; Jayanthi et al., 2016), osmium (Suntharalingam et al., 2013), and iridium (Cao et al., 2013) complexes have been shown to activate a number of ICD markers involving ROS production, unfolded protein response (UPR) and ER stress. Considering the overexpression of the ROS, protein damage, UPR and ER stress also as mechanisms of action of gold compounds, it is reasonable to believe that there are more types I and II gold-based ICD inducers. Auronafin induced a concentration-dependent increase of cellular ROS in human lung cancer cell lines (A549) (Hou et al., 2018). Alkynyl gold(I) complex was able to disrupt mitochondrial normal function and induced the production of ROS, which triggered necroptosis in the colorectal adenocarcinoma (Caco-2) cell line (Marmol et al., 2017). Organometallic gold(III) complexes induced ER-stress-related apoptosis as well as pro-death autophagy in lung cancer (A549) cells, allowing lower toxicity and better antitumor activity in murine tumor model comparing with cisplatin (Huang et al., 2018). It has been reported that accumulated lethal DNA double strand breaks due to the increase of ROS render ovarian cancer cells more sensitive to auranofin in a BRCA1-deficient background. Furthermore, the antioxidant N-acetyl cysteine (NAC) protected BRCA1-deficient cells from auranofin-induced DNA damage and apoptosis (Oommen et al., 2016). Strikingly, the ICD induction ability of the experimental and clinically used gold compounds has not been systematically and fully identified so far.

### Gold Compounds and Immune Checkpoints

Immune checkpoints can negatively regulate T-cell immunity. The discovery of immune checkpoint inhibitors has opened up new clinical possibilities for anticancer immunotherapy. Among them, programmed death receptor 1 (PD-1) and cytotoxic T lymphocyte antigen 4 (CTLA-4) are the most commonly studied. PD-1 is mainly expressed on the surface of activated T cells, B cells, as well as NK cells. The activation of PD-1 suppresses the phosphatidylinositol 3-kinase (PI3K)/Akt signaling pathway, leading to the inhibition of survival and proliferation of T cells (Pedoeem et al., 2014). PD-1 has two main ligands: PD-L1 and PD-L2, which are involved in inducing T-cell exhaustion. Targeting PD-L1 in some early clinical cancer studies has shown beneficial effects, which has been welcomed by researchers. PD-L1 is widely expressed and can be detected in both hematopoietic cells and non-hematopoietic healthy tissue cells. Numerous studies have confirmed that the expression of PD-L1 gene is controlled by inflammatory signaling. Many soluble factors secreted by immune cells have been described as inducers of PD-L1 in the past few years. INF can regulate the expression of PD-L1 in many types of tumors and immune cells, as well as healthy tissues (Brown et al., 2003). The binding of IFN-γ to its receptor activates the classical JAK/STAT signaling pathway, inducing an increase in the expression of a series of transcription factors, known as interferon-response factors (IRFs). Among these factors, IRF1 is prerequisite to the IFN-γ-mediated upregulation of PD-L1 (Lee et al., 2006). In addition, studies have reported that the up-regulation of PD-L1 depends on TLR4/STAT1 pathway, while the expression of PD-L2 depends on IL-4/STAT6 pathway. As discussed above, it makes sense for immunotherapy considering that STAT signaling is key to activating immune checkpoint molecules such as PD-L1 (Loke and Allison, 2003). It was reported that auranofin inhibited IL-6-induced activation of JAK2/STAT3 signaling pathway and activation of NF-κB in human multiple myeloma cell line (U266, RPM8226, and IM9). The phosphorylation of STAT3 was inhibited by auranofin through IL-6, leading to down-regulation of the anti-apoptotic proteins Mcl-1 and apoptosis of myeloma cells (Nakaya et al., 2011). In some similar experiments, auranofin also blocked the IL-6-mediated JAK1/STAT3 signaling pathway in HepG2 human hepatoma cells and primary cells (i.e., human umbilical vein endothelial cells, fibroblast-like synoviocytes and rat astrocytes) (Kim et al., 2007). In human breast cancer cells (MDA-MB 231), STAT3 phosphorylation and telomerase activity were also decreased by auranofin, but N-acetyl-L-cysteine (a scavenger of ROS) pretreatment restored STAT3 and telomerase activity (Kim et al., 2013). What's more, in a recent study, Raninga et al. for the first time found that auranofin could increase CD8 ^+Ve^ T- cells tumor infiltration and upregulate the expression of PD-L1 by an ERK1/2-MYC-dependent manner in mice models of triple-negative breast cancer (TNBC). Their study provides a novel combination of cancer therapy using auranofin in combination with anti-PD-L1 targeting therapy, which has limited clinical efficacy in TNBC patients with monotherapy (Raninga et al., 2020). As far as we know, gold derivatives have no effect on other immune checkpoints. Although there is very little research on the direct effects of gold compounds on immune checkpoints, the evidence above suggests a close relationship between gold compounds and the regulation of immune checkpoints.

## Gold Drugs Combined With Anticancer Immunotherapy

Metal-based anticancer drugs are widely used in the clinical treatment of various types of tumors. This situation shows that the systemic anticancer treatment based on metal drugs has high activity and quality, and it is urgent to guide the development of anticancer metal drugs by multidisciplinary approaches. Based on the promising prospect of the anticancer activity of gold derivatives, a large number of articles have been published in recent years (Yeo et al., 2018; Zhang et al., 2018b; Englinger et al., 2019; Mora et al., 2019). By strengthening our understanding of the complex effects of gold compounds on the host immune system, we can develop them better. Immunotherapy is a great revolution in the history of tumor treatment. Tumor immunotherapy has made a series of progress in recent years, which has changed the treatment pattern of many cancers and plays a very important role in the current scientific research. Compared with traditional chemotherapy and targeted therapy, the essential difference is that immunotherapy targets the immune cells and strengthens the immune system, instead of impairing the immune system. As a result, it can treat various types of tumors and is less prone to drug resistance. As we described before, the apparent immunomodulatory effects of gold compounds *in vivo* and *in vitro* support the hypothesis that these antitumor drugs might be ideal combination partners for immunotherapeutic interventions. Among the various immunotherapeutic strategies, the treatment based on immune checkpoint inhibitors is a highly efficient anticancer therapy and has shown unparalleled efficacy in patients with advanced cancer. A central challenge in modern systemic cancer treatment research is to identify and develop personalized and multitargets combination therapy strategies to improve the efficacy of modern immunotherapies. In Raninga's research, auranofin combined with immune checkpoint PD-L1 inhibitor achieved excellent anti-tumor efficacy in breast cancer cell lines and in mice, demonstrating the promising prospect of combined immunotherapy based on gold derivatives (Raninga et al., 2020). Such experiments are difficult to carry out in humans, largely hindering the exploration of specific mechanisms of gold compounds and their complex interactions with immune system. More attempts should be made to explore the clinical efficacy and prognosis of gold derivatives in combination with other checkpoint inhibitors, CAR-T cell therapy, monoclonal antibodies and molecular-targeted agents.

## The Adverse Effects of Gold Drugs

The immune system plays an important role in the side effects of gold compounds. Interestingly, the effects of gold compounds on the immune system can be broadly divided into two categories. On the one hand, some adverse effects can be related to immune-stimulating reactions, such as various forms of dermatitis, glomerulonephritis, and enteritis. The most common toxicity of gold compounds includes allergic reactions of skin and mucous membranes, such as skin rash, pruritis, contact dermatitis, stomatitis, as well as conjunctivitis. Some patients developed proteinuria during treatment, and the most common renal histologic lesion was membranous glomerulonephritis (Tonroth and Skrifvars, 1974). There are no detailed cases of long-term severe or permanent renal damage caused by gold compounds. Pulmonary toxicity is rare and mainly in the form of diffuse interstitial lung disease when treated with injectable gold compounds. The presence of cholestatic jaundice and acute enterocolitis is also associated with injectable gold compounds. On the other hand, also immunosuppression reactions may occur, such as the impairment of macrophages, T and B cells, aplastic anemia (Hansen et al., 1985), and bone marrow suppression (Yan and Davis, 1990). Bone marrow aplastic anemia is the most severe, but relatively rare. Overall, the toxicity of early injectable gold compounds was greater than that of oral gold compounds. Furthermore, we can observe that gold compounds combine with different ligands to produce different toxic reactions, so we can design more optimized ligands to reduce side reactions of gold compounds. Among these, N-Heterocyclic carbine (NHC) ligands have shown very promising results in recent years because they fully meet the preconditions for efficient drug design and rapid optimization (Zhang et al., 2018b; Mora et al., 2019).

## Conclusions

In this review article, we systematically summarize the unique modulatory effects of gold compounds on antitumor immunity, including the enhancement of antigenicity and immunogenicity of tumor cells, the effects on immune cells and immune checkpoints, as well as the induction of ICD. Elucidating these important issues will greatly improve the identification and development of anticancer gold derivatives. In the future, gold compounds may have great application prospects in the combination therapy, potentiating the efficacy of targeted therapies and immunotherapy, especially immune checkpoint inhibitors therapy. Further studies are required to understand the detailed mechanisms of the regulation of anticancer immune response by gold derivatives in order to promote their clinical application.

## Author Contributions

SW and HL: had full access to all of the data in the study and take responsibility for the integrity of the data and the accuracy of the data analysis, concept, design, and critical revision of the manuscript for important intellectual content. SY and ML: acquisition, analysis, or interpretation of data and administrative, technical, or material support. SY and SW: drafting of the manuscript. All authors contributed to the article and approved the submitted version.

## Conflict of Interest

The authors declare that the research was conducted in the absence of any commercial or financial relationships that could be construed as a potential conflict of interest.
